# Theoretical and simulation studies on voltage-gated sodium channels

**DOI:** 10.1007/s13238-015-0152-6

**Published:** 2015-04-17

**Authors:** Yang Li, Haipeng Gong

**Affiliations:** MOE Key Laboratory of Bioinformatics, School of Life Sciences, Tsinghua University, Beijing, 100084 China

**Keywords:** voltage-gated sodium channels, molecular dynamics simulation, ion permeation, ion selectivity, voltage gating

## Abstract

Voltage-gated sodium (Na_v_) channels are indispensable membrane elements for the generation and propagation of electric signals in excitable cells. The successes in the crystallographic studies on prokaryotic Na_v_ channels in recent years greatly promote the mechanistic investigation of these proteins and their eukaryotic counterparts. In this paper, we mainly review the progress in computational studies, especially the simulation studies, on these proteins in the past years.

## **INTRODUCTION**

As one of the fundamental elements in the membrane of excitable cells, voltage-gated sodium (Na_v_) channels are critical in the generation and propagation of electrical signals in both nerves and muscles (Hille, 2001), and therefore have become therapeutic targets for many neurological disorders, including epilepsy, migraine, neurodegenerative diseases and neuropathic pain (Clare et al., 2000; Dib-Hajj et al., 2010; Mantegazza et al., 2010). Na_v_ channels are members of the voltage-gated ion channels (VGICs), a superfamily of proteins that allow the cross-membrane permeation of various ions under the control of the cross-membrane voltage (Ertel et al., 2000; Catterall et al., 2005; Gutman et al., 2005). Many VGIC superfamily members, particularly the voltage-gated cation channels (VGCCs) that control the transport of Na^+^, K^+^ and Ca^2+^ ions (named as Na_v_, K_v_ and Ca_v_ channels respectively), adopt similar structural topologies and mechanisms (Hille, 2001; Catterall et al., 2005). The physiological roles and mechanisms of VGCCs have been extensively studied in history, even in the absence of structural information. Among these previous researches, we want to particularly mention two famous biophysical models which have been generally accepted because of their agreement with enormous experimental observations: the Hodgkin-Huxley model and the knock-on model. The former perfectly explains the relationship between VGCCs and the electrical signaling in excitable cells (Hodgkin and Huxley, 1952) while the latter describes the mechanism of ion permeation in these channels (Hodgkin and Keynes, 1955). Moreover, the electrophysiological studies over the past half of century indicate two key characteristics of VGCCs: ion selectivity and voltage gating (Favre et al., 1996; Sontheimer et al., 1996; Sun et al., 1997; Catterall, 2000), both of which are essential for the normal electrical signaling. The former requires the channel to selectively allow the permeation of its target cation in the presence of various other ions, and the latter enables the channel to open and close in response to the variation of cross-membrane voltage.

**Figure 1:**
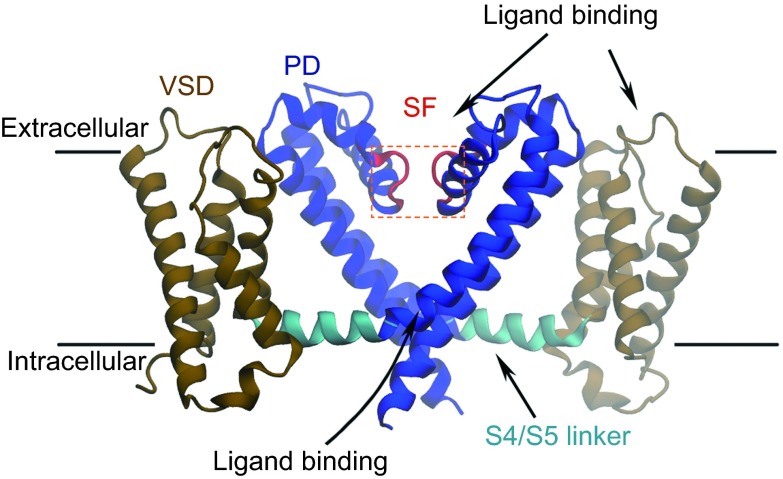
**The structural organization of VGCCs**. Only the opposing two TDs are shown to facilitate visual recognition. The PD (purple) and VSDs (brown) are connected by a half helix (cyan) between TMs S4 and S5. The SF region within the PD is colored in red. Ligands can bind at both PD and VSDs, as shown by the black arrows

In the late 1990s, the potassium channels became the breakthrough point for the structural studies on VGICs (Doyle et al., 1998; Jiang et al., 2002; Jiang et al., 2003; Long et al., 2005; Long et al., 2007). Unlike the K_v_ channels that are assembled from four identical subunits, the corresponding four structural units are joined to form a single polypeptide of more than 2000 amino acid residues in eukaryotic Na_v_ channels, a structural organization that brings tremendous additional difficulty on their structural determination. The earliest Na_v_ structure was purified from the electric organ of the eel *Electrophorus electricus* and was determined by helium-cooled cryo electron microscopy (Sato et al., 2001) with low resolution (19 Å). Almost at the same time, a prokaryotic Na_v_ channel was found to possess similar characteristic of ion selection with its eukaryotic counterpart (Ren et al., 2001). This observation encouraged crystallographic studies on the prokaryotic Na_v_ channels, since they follow the same homotetrameric architecture as potassium channels (Yu and Catterall, 2004; Payandeh and Minor, 2015). A large family of bacterial Na_v_ channels were identified and the high-resolution structures of several orthologs were sequentially determined in recent years (Payandeh et al., 2011; McCusker et al., 2012; Payandeh et al., 2012; Zhang et al., 2012; Shaya et al., 2014). According to the structural insights from these prokaryotic homologs, all Na_v_ channels are supposed to consist of four transmembrane domains (TDs), each of which adopts a unique amino acid sequence (Catterall et al., 2005). Every TD contains six transmembrane helices (TMs), which are named from S1 to S6 sequentially. TMs S1–S4 form the voltage-sensing domain (VSD) that is responsible for perceiving the changes of cross-membrane voltage, while TMs S5–S6 as well as the intervening P-loops and half helices from the four TDs jointly constitute the pore domain (PD) that allows the ion permeation (Fig. 1). Particularly notably, the P-loop from each TD contribute 6–7 residues to form a geometrically constricted region at the extracellular side of the PD, which is mainly responsible for ion selection according to mutagenesis analysis (Favre et al., 1996; Schlief et al., 1996; Sun et al., 1997) and is therefore named as the selectivity filter (SF).

Despite the success in the structural biology, the mechanistic illustration on the ion selection, voltage gating and ion permeation for Na_v_ channels are still lacking, since each crystal structure only reflects one static conformation and therefore cannot describe the structural dynamics that is required for the proper functioning of proteins. As a complementary tool, computer simulations, particularly the molecular dynamics (MD) simulations and the associated theoretical calculations, can overcome such deficiencies and have therefore become essential tools in the investigation of a wide range of chemical and biological systems (Karplus and Petsko, 1990; Karplus and McCammon, 2002). As an example, the pioneering simulation studies on potassium channels performed by Roux and coworkers even anticipated some results that were validated in the subsequent crystallographic studies (Roux and MacKinnon, 1999; Berneche and Roux, 2001). In the past decade, progress in hardware development, improvement in simulation methodologies and refinement of interaction potentials have rendered the possibility to model increasingly complex processes that were intractable previously (Karplus and Kuriyan, 2005). Nowadays, with the maturation of simulation protocols (Phillips et al., 2005; Hess et al., 2008; Brooks et al., 2009; Case et al., 2012), MD simulations about ion channels have gradually reached satisfying agreement with the experimental data (Allen et al., 2006; Gordon et al., 2013), although most of the prominent achievements were obtained for the potassium channels (Roux and MacKinnon, 1999; Shrivastava and Sansom, 2000; Berneche and Roux, 2001; Noskov et al., 2004; Noskov and Roux, 2006; Jensen et al., 2010; Delemotte et al., 2011; Jensen et al., 2012). With the successful determination of bacterial Na_v_ structures in the past years, several groups have conducted MD simulations and proposed models to illustrate the molecular mechanism of Na_v_ channels. In this review, we will go over the progress of these simulation studies in the following four categories: ion binding and permeation, ion selectivity, voltage gating and ligand binding.

## **ION BINDING AND PERMEATION**

Quickly after the first Na_v_ structure (Na_v_Ab) was unveiled in 2011, an MD simulation was performed to investigate the interaction between Na^+^ ions and this protein (Carnevale et al., 2011). The Na_v_Ab structure is believed to represent a pre-active state, since its PD is completely closed to cytosol although the four VSDs have reached the active (or “up”) state (Payandeh et al., 2011). The overall protein structure was stable during the 140 ns equilibrium simulation, with the cytoplasmic entrance of the PD remaining closed to the cytoplasmic ions. However, the periplasmic Na^+^ ions quickly entered the SF and bound at two favorable sites, which were named as S_HFS_ and S_CEN_ respectively. Interestingly, Na^+^ ions bound at these sites are coordinated by the oxygen atoms from both protein and water. Such an asymmetric binding pattern with incomplete dehydration is different from the observations in the potassium channels where the K^+^ ions are completely dehydrated and form strong coordination with eight symmetrically positioned carbonyl oxygen atoms of the protein (Zhou et al., 2001).

**Figure 2:**
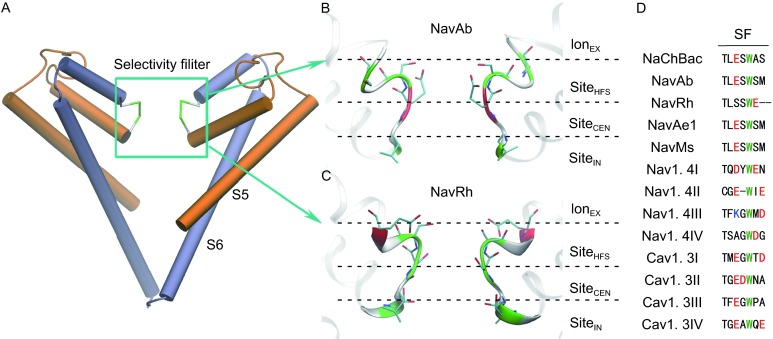
**The ion binding sites identified in the SF of Na**_**v**_ **channels**. (A) The SF region is labeled by a cyan frame. (B) The schematic ion binding sites in Na_v_Ab. (C) The schematic ion binding sites in Na_v_Rh. (D) Aligned amino acid sequences for the SF region in various prokaryotic Na_v_ channels as well as eukaryotic Na_v_ and Ca_v_ channels. The four structural units in the eukaryotic channels are labeled as I, II, III and IV, respectively

The unique ion binding behavior of the sodium channels was later confirmed by a series of subsequent simulations (Corry and Thomas, 2012; Furini and Domene, 2012; Qiu et al., 2012; Chakrabarti et al., 2013; Boiteuxa et al., 2014). According to both single-ion and multi-ion profiles of the potential of mean force (PMF) evaluated along the ion permeation pathway within the SF (Corry and Thomas, 2012; Furini and Domene, 2012; Qiu et al., 2012), several favorable ion binding sites were rigorously identified and were named as S_HFS_, S_CEN_ and S_IN_ in the order from the periplasmic entrance to the central cavity (Fig. 2). Moreover, in all equilibrium simulations, the protein structures were stable and the periplasmic Na^+^ ions quickly entered the unoccupied SF together with coordinated water molecules. The ion initially arrived at the site S_HFS_ and then permeated further to the more favorable site S_CEN_, where the ion could either transit backward to S_HFS_ intermittently or move forward to S_IN_ and even the central cavity within the nanosecond timescale of simulations (Furini and Domene, 2013). The site S_HFS_ was named to reflect the high field strength, since it is surrounded by the carboxylate groups from four Glu residues (or the EEEE motif). Notably, this site is of great interest since the corresponding residues in all eukaryotic homologs are highly conserved (as Asp, Glu, Lys and Ala respectively, or the DEKA motif) and have been reported as the key residues to determine the ion selectivity (Catterall, 2000). Despite the electrostatic repulsion between the acidic residues at S_HFS_ in Na_v_Ab, the simulations indicated that at least three of the Glu residues should be deprotonated in order to maintain the efficient ion permeation (Boiteuxa et al., 2014; Furini et al., 2014). In the absence of cations, these Glu side groups uniformly point towards the periplasm to minimize the unfavorable repulsion (Chakrabarti et al., 2013). After the cation arrives at this site, one or two Glu side groups may swing to a partially downward orientation (Boiteuxa et al., 2014) to facilitate ion diffusion.

With the determination of more Na_v_ structures, some groups began to simulate these orthologous proteins. The Na_v_Rh structure was claimed to represent the inactive state, since the side chains of four Ser residues occlude the ion permeation pathway in the SF of the crystal structure (Zhang et al., 2012). In the simulation, however, the collapsed SF region quickly became ion accessible by the self-adjustment of side chains (Zhang et al., 2013). Although the Na_v_Ms crystal structure lacks the VSDs, its PD exhibits a continuous pore that geometrically allows ion permeation (McCusker et al., 2012), which enables the evaluation of conductance of the channel from MD simulations. The electric current and the conductance estimated in the subsequent simulations agreed well with the values obtained from electrophysiological experiments (Ulmschneider et al., 2013), which further reinforces the power of MD simulation in the study of ion channels. In spite of the minor difference in details, simulations on both orthologs echoed the observations in the simulations of Na_v_Ab: the SF contains multiple ion binding sites and cations bind in an asymmetrical manner with incomplete dehydration (Ulmschneider et al., 2013; Zhang et al., 2013).

After the identification of ion binding sites, the ion permeation patterns were investigated in numerous simulations (Carnevale et al., 2011; Furini and Domene, 2012; Qiu et al., 2012; Chakrabarti et al., 2013; Ke et al., 2013; Stock et al., 2013; Ulmschneider et al., 2013; Zhang et al., 2013; Bagnéris et al., 2014; Boiteuxa et al., 2014). Nearly all studies indicated the presence of weak coupling between the 2–3 permeating Na^+^ ions accommodated within the SF. This permeation mechanism is different from that of the potassium channels where K^+^ ions move in a highly concerted manner, and is therefore proposed as a loosely coupled knock-on mechanism. Since the closed conformation of Na_v_Ab hinders the observation of continuous ion transport, Klein and Treptow as well as their coworkers modeled its open conformation based on a homologous structure from the potassium channel (Amaral et al., 2012). Their subsequent simulations suggested that Na^+^ ions permeate in pairs within the SF for most of the inward ion flows and that these ion pairs transit among five major configurations (Stock et al., 2013). According to the PMF calculation, the energy barrier for the overall permeation process of such a two-ion mode is ~3 kcal/mol (Stock et al., 2013). However, a third ion could participate in the outward ion flows, which leads to an average occupancy of 2.3 ions within the SF (Stock et al., 2013). Similarly, Wallace and coworkers simulated the continuous inward ion conduction in the open Na_v_Ms structure with a constant electric field applied across the membrane, and found that on average 1.8 Na^+^ ions are involved in the ion permeation within the SF (Ulmschneider et al., 2013). These simulations supplemented the weakly coupled knock-on mechanism with molecular details. Apart from the weakened coupling between permeating ions, the water flux through the sodium channels is uncorrelated with ion flux (Ulmschneider et al., 2013), in sharp contrast to the potassium channels where the rate of ionic flow limits the maximum water flux through the channel (Saparov and Pohl, 2004; Jensen et al., 2010).

## **ION SELECTIVITY**

Selective conduction of Na^+^ ions is mainly conferred by the SF region, which is highly conserved among all known Na_v_ channels (Catterall et al., 2005). The mechanism of ion selection has been theoretically investigated decades before, in the absence of any crystal structures. Eisenman ascribed the ion selection to the field strength of the ion-coordinating groups and predicted that partitioning of the better solvated Na^+^ ions into the SF of potassium channel is unfavorable (Eisenman and Horn, 1983; Hille, 2001). With the great success of crystallographic studies on ion channels, the potassium channels soon became a good prototype for comprehending the mechanism of selectivity for the various ions. The SF regions of potassium channels are geometrically confined so that the ions have to be completely dehydrated when entering this region. The de-solvation of cations is energetically compensated by the coordination with symmetrically distributed carbonyl oxygen atoms donated by the P-loop residues. Naïve structural analysis suggested that the K^+^ ions are snugly coordinated by eight carbonyl oxygen atoms at the most favorable distance and that both the number of coordinating groups and the coordinating distances in the crystal structure render the site’s preference of K^+^ binding (Doyle et al., 1998; Zhou et al., 2001). Since the structural dynamics were completely neglected in such naïve analysis, Roux and coworkers then conducted MD simulations on the KcsA channel and evaluated the difference between ion binding affinities using the free energy perturbation (FEP) method (Noskov et al., 2004). According to their calculations, the electrostatic repulsion between the carbonyl groups partially restrains the structural fluctuation of the ion binding sites within the SF, and therefore prevents coordinating groups from interacting with Na^+^ ions at shorter and more favorable distances (Noskov et al., 2004). In numerous following studies, people tended to explain the ion selectivity in terms of the number and field strength of coordinating groups by assuming that they form a homogeneous coordinating environment (Noskov et al., 2004; Bostick and Brooks, 2007; Fowler et al., 2008).

The emergence of high-resolution crystal structures for the sodium channels broke the theoretical foundation of such analysis, both because the sodium channels use sidechain atoms rather than the backbone carbonyl groups to coordinate ions and because the ion coordinating groups are heterogeneously distributed. Furini and Domene evaluated the 1D PMF profiles of Na^+^ and K^+^ ions along the permeation pathway in the SF of Na_v_Ab. Although the two types of ions bind at similar sites (S_CEN_ and S_HFS_), the free energy barrier for K^+^ permeation is 2–3 kcal/mol higher, which relatively disfavors the K^+^ conduction in kinetics. Such a small difference in the free energy barrier agrees with the mild Na^+^/K^+^ selectivity of the Na_v_Ab channel (Furini and Domene, 2012). Corry and Thomas proposed an alternative hypothesis according to their simulations on Na_v_Ab, by assuming that a water molecule bridged between the ion and the carboxylate group of one Glu residue at the site S_HFS_ confines the pore size at this site and therefore prohibits the passage of the larger K^+^ ions (Corry and Thomas, 2012). The subsequent studies examined the equilibrium behavior of Na_v_Ab by performing MD simulations in microseconds and reported more evenly distributed free energy landscape for Na^+^ ions (Chakrabarti et al., 2013; Boiteuxa et al., 2014). The free energy barrier between various binding modes are effectively reduced by the downward swinging of the Glu side groups at the site S_HFS_, a phenomenon that was neglected in previous simulations with a large negative internal voltage applied across the membrane (Stock et al., 2013; Ulmschneider et al., 2013). As pointed out in previous theoretical studies, reasonable structural fluctuations are required for the mechanistic understanding of ion selection (Allen et al., 2004), and the interplay between the attractive ion-ligand and repulsive ligand-ligand interactions jointly govern the Na^+^/K^+^ selectivity in the flexible binding sites (Noskov and Roux, 2006).

Besides the interference from the outward flow of cytoplasmic K^+^ ions, the periplasmic Ca^2+^ ions may compete with Na^+^ ions for the inward permeation through the sodium channels. Therefore, the selection of Na^+^ vs. Ca^2+^ ions in Na_v_ channels is of particular interest, since Ca^2+^ ions frequently act as secondary messengers and their cross-membrane leaking may cause cell disorder. Interestingly, eukaryotic Ca^2+^ channels have the EEEE motif at the constriction site (similar to Na_v_Ab) but they strongly prefer Ca^2+^ over Na^+^ ions (Sather and McCleskey, 2003). Dudev and Lim investigated this phenomenon using a reduced model system and proposed the protein matrix as the decisive factor for Ca^2+^ vs. Na^+^ selectivity in the SF of calcium channels (Dudev and Lim, 2012). On the other hand, simulations on Na_v_Ab suggested that Ca^2+^ translocation lacks the knock-on mechanism for efficient permeation although they can favorably bind at sites S_CEN_ and S_HFS_ (Ke et al., 2013). In the SF of Na_v_Rh, the site S_HFS_ is composed of four Ser residues and Glu residues are located three residues away on the periplasmic side to constitute a site called Ion_EX_ (Fig. 2C and 2D). Our simulations on Na_v_Rh suggested that the extremely strong binding preference of Ca^2+^ ions at Ion_EX_ renders a huge free energy barrier (~10 kcal/mol) which prevents the Ca^2+^ ions from further moving towards the site S_CEN_ (Zhang et al., 2013), an explanation consistent with the experimental observation that periplasmic Ca^2+^ ions block the Na^+^ flux in Na_v_Rh (Zhang et al., 2012).

Note that most of these proposed mechanisms for ion selectivity are applicable to the prokaryotic Na_v_ channels only, since eukaryotic channels have a highly conserved DEKA motif rather than the EEEE motif at the position equivalent to the site S_HFS_ of Na_v_Ab and since the DEKA motif has been identified to be the major determinant of the ion selection in most eukaryotic Na_v_ channels (Favre et al., 1996; Catterall, 2000). Lipkind and Fozzard generated a homology model for the eukaryotic Na_v_ channels in the lack of any available structures for sodium channels. Based on the extensive experimental data and their MD simulations on this structural model, they proposed that electrostatic competition between the alkali cations and the residue pair of Glu and Lys (E and K in the DEKA motif) finely tuned the preference of ion binding at S_HFS_ towards Na^+^ ions in eukaryotic Na_v_ channels (Lipkind and Fozzard, 2008). Subsequently, Dudev and Lim investigated the factors controlling Na^+^/K^+^ selectivity in sodium channels using reduced models and proposed that the architecture, chemical composition and physicochemical properties of the SF in eukaryotic sodium channels jointly contribute to the Na^+^/K^+^ selectivity (Dudev and Lim, 2010). After the structural determination of Na_v_Rh, we modeled the structure of eukaryotic Na_v_ channels by mutating the Ser residues at the constriction site in Na_v_Rh to form a DEKA motif. By conducting MD simulations on this structural model and evaluating the 2D PMF profiles of ion binding at this site, we successfully identified the essential roles of Lys and Asp/Glu in determining the Na^+^/K^+^ selectivity. The positively charged Lys repels the cation to bind at the position sandwiched between the two carboxylate groups of Asp and Glu, a location where the cations are coordinated by these carboxylate oxygen atoms at short distances that are favorable for Na^+^ binding (Xia et al., 2013). In a recent work, Dudev and Lim discussed the evolution of eukaryotic VGCCs with their ion preference converted from Ca^2+^-favored to Na^+^-selective (Dudev and Lim, 2014).

## **VOLTAGE GATING**

VGCCs needs to open and close in response to the variation of cross-membrane potentials and the perception on voltages is accomplished by the VSDs, which are composed of TMs S1–S4 in each TD (Yu and Catterall, 2004). Once the changes in voltage is sensed by the VSDs, these domains experience large-scale conformational change among the resting, active and inactive states, which subsequently triggers the PD structure to transit between the open and closed states (Hodgkin and Huxley, 1952; Isacoff et al., 2013). The combination of the conformational changes of both the VSD and PD following the voltage change is called the voltage gating, which is indispensable for the initiation and propagation of the action potentials in mammals. The TM S4 has been supposed to be the key element for voltage sensing. This helix carries 4–5 highly conserved positively charged residues (called gating charges), which tend to move in the extracellular direction and generate gating current when the membrane is depolarized (Kuzmenkin et al., 2004; Blanchet and Chahine, 2007; DeCaen et al., 2009; Yarov-Yarovoy et al., 2012; Zhang and Yan, 2013). Among the numerous models proposed to describe the movement of S4 helix, the most famous one is the sliding-helix mechanism (Catterall, 2010), which stated that in response to the membrane depolarization, the S4 helix slides within the membrane towards the extracellular side by the sequential formation and break of ion pairs composed of the gating charges on S4 and acidic residues on S1–S3 (Vargas et al., 2012). A conserved Phe residue in the hydrophobic constriction site (HCS) and two negative charges have been implicated as a gating charge transfer center in K_v_ channels (Tao et al., 2010). Nevertheless, the VSDs of the VGCCs frequently form an hourglass-shaped structure within the membrane that essentially functions as a voltage-dependent Arg side-chain transporter (Payandeh and Minor, 2015).

The structural determination of prokaryotic and eukaryotic K_v_ channels (K_v_AP and K_v_1.2/K_v_2.1 chimera channel) (Jiang et al., 2003; Long et al., 2007) stimulated the interests of computational biologists to simulate their voltage gating process. Delemotte et al. conducted MD simulations on the VSD of K_v_1.2 and analyzed effect of residue mutation on the gating behavior (Delemotte et al., 2010). Subsequently, Jensen et al. simulated the whole protein (VSD + PD) for milliseconds and directly observed the complete protein conformational change in the hyperpolarization and depolarization conditions (Jensen et al., 2012). They then proposed a detailed mechanistic model to illustrate the molecular events occurred during the inactivation and activation processes of K_v_ channels. In a recent work, Delemotte et al. found the reaction pathway for the first step of structural transition during the activation of the VSDs in K_v_1.2 and rigorously evaluated the PMF profile along the reaction coordinate (Delemotte et al., 2015). Their calculations were consistent with the phenomenological models adopted to illustrate the observations and measurements in physiological experiments.

The voltage gating in Na_v_ channels has been lagging behind for a long time, because of the late emergence of high-resolution structures. In the absence of the structural information for Na_v_ channels, Catterall and coworkers took the K_v_AP and K_v_1.2 structures as templates and used the Rosetta program to model the structure basis for gating charge movement in the VSD of the NaChBac channels during activation (DeCaen et al., 2008; DeCaen et al., 2009; Yarov-Yarovoy et al., 2012). Since the structural determination of Na_v_Ab, which is believed to reflect a pre-active conformational state (Payandeh et al., 2011), Klein and Treptow as well as their coworkers have conducted a series of simulations to investigate the gating mechanism of Na_v_ channels. Benefited from their previous success in the simulation of K_v_ channels (Treptow and Tarek, 2006; Delemotte et al., 2011), they modeled the activated-open and resting-closed states of Na_v_Ab using the experimental and simulated conformations of K_v_1.2 as templates, and simulated the structural transition of Na_v_Ab from the activated-closed state (in the crystal structure) towards these two states (Amaral et al., 2012). In another work, they modeled the structure of NaChBac based on Na_v_Ab and simulated the conformational change of this model structure upon activation (Barber et al., 2012).

## **LIGAND BINDING**

The identification of the different subtypes in pathophysiology of Na_v_ channels has provided a rational basis for selective intervention in clinic treatment and some drugs have shown therapeutic value in Na_v_ channelopathies (Ragsdale and Avoli, 1998; Mantegazza et al., 2010; Payandeh et al., 2012; Bagal et al., 2013; McCormack et al., 2013; Bagnéris et al., 2014). Many pathogenic Na_v_ channels have been intensively investigated by *in vitro* mutagenesis and electrophysiological experiments. On the other hand, an array of highly potent and selective neurotoxins have proven the lethal effects by destroying the normal functional modulation of Na_v_ channels, including pore blockage, over-stabilization of the opened pore as well as alteration on the movement of voltage sensors and on the gating mechanism (Hille, 2001; Catterall et al., 2005; Dib-Hajj et al., 2010; Stevens et al., 2011; Knapp et al., 2012; Moreau et al., 2014; Thottumkara et al., 2014; Kalia et al., 2015; Payandeh and Minor, 2015).

Computational studies, including MD simulations, Brownian dynamics and molecular docking, have become new tools to study the roles of various subtypes of Na_v_ channels in pathophysiology at an atomistic level (Gordon et al., 2013). Even before the systematic crystallographic studies on VGICs, Lipkind and Fozzard had developed a reasonable molecular model to supposedly describe the binding pockets for tetrodotoxin (TTX) and saxitoxin (STX) in eukaryotic Na_v_ channels (Lipkind and Fozzard, 1994). At present, some toxin-channel interactions can be investigated at atomistic levels using the available high-resolution structures of the prokaryotic Na_v_ channels, by considering that the SF region is structurally stable during the evolution of Na_v_ channels (Tikhonov and Zhorov, 2012). Although some bacterial Na_v_ channels are resistant to TTX (Ren et al., 2001), MD simulations indicated that μ-conotoxins is effective in inhibiting the Na_v_Ab structure (Chen and Chung, 2012a), which was further confirmed in the electrophysiological experiment (Stevens et al., 2012). In the subsequent simulations, Chen and Chung identified the functional surface of β-toxins to interact with Na_v_ channels (Chen and Chung, 2012b). This computationally predicted function surface appeared to overlap with that of α-toxins determined experimentally (Gordon et al., 2007). In a later work, they used MD simulations to investigate the interaction between TTX and a model structure of Na_v_1.4, and found that TTX may occlude the channel entrance by forming a network of hydrogen bonds in the outer lumen of the SF (Chen and Chung, 2014), consistent with the experimental evidence that the charged outer ring of the SF is critical in the TTX blockage (Penzotti et al., 1998). Recently, Allen and coworkers conducted microseconds of MD simulations to comprehensively analyze the interactions between lipophilic drugs (benzocaine and phenytoin) and Na_v_Ab (Boiteuxa et al., 2014). They finally identified two different drug-access pathways, which may provide insight into mechanistic studies on the Na_v_ channel inhibition and may assist future drug development.

## **OUTLOOK**

Computer simulations have experienced impressively rapid development in the past decade. On one hand, the conventional simulation time has risen from nanoseconds to microseconds or even milliseconds, due to the improvement in both computer hardware and algorithms. On the other hand, numerous new methods have been developed to facilitate overcoming the energetic barrier and therefore more efficient sampling in the conformational space (Piccinini et al., 2008). We could only list a few here: accelerated molecular dynamics (Hamelberg et al., 2004) and metadynamics (Laio and Gervasio, 2008) to enhance the conformational sampling, the STRING method (Maragliano et al., 2006) to find the minimal free energy path (MFEP) during the structural transition, the orthogonal space random walk (Zheng et al., 2008) and adaptive umbrella sampling (Bartels and Karplus, 1998) to allow the identification of an ensemble of reaction pathways and reliable free-energy estimation, etc. Improvements in both facets jointly enable the observation of large-scale conformational changes that were unmanageable previously, particularly the voltage gating process of Na_v_ channels. Moreover, simulations with the polarizable force field (Jiang et al., 2011) has been available to consider the charge transfer between ion and proteins during the transport process, which could further improve the accuracy of observations and calculations on the ion selectivity and the ion permeation mechanism. The molecular mechanisms of Na_v_ channels are expected to be better illustrated in the future with these new techniques.

Despite the above progresses, the simulation studies highly depend on the crystallographic achievement. In the absence of high-resolution structures, the simulations have to start from a modeled structure, which eventually weakens the reliability of the simulation observations. The simulation studies on Na_v_ channels are awaiting the determination of more structures, especially eukaryotic structures. The eukaryotic Na_v_ channels have the unique DEKA motif in the SF that is absent in the prokaryotic counterparts. Consequently, the molecular mechanisms of ion permeation and ion selection in eukaryotic Na_v_ channels cannot be accurately illustrated from the simulations on their prokaryotic homologs. On the other hand, the structural determination of other conformational states, especially the resting state conformation, will greatly facilitate the simulation studies on the voltage gating mechanism. All currently available structures have their VSDs maintained at the “up” position. Therefore, a resting state conformation is required for a comprehensive understanding on the overall cycle of conformational change. The presence of representative structures for both the active and resting states will greatly improve the efficiency of free energy analysis for the activation and de-activation processes. In addition, the computational drug development on Na_v_ channels requires the determination of structures from higher-level organisms, especially mammals. In summary, with more structures of Na_v_ channels solved in the near future, computer simulations are expected to play more important roles in connecting the molecular details and macroscopic experimental measurements for these channels.
